# Multiple independent origins of a protease inhibitor resistance mutation in salvage therapy patients

**DOI:** 10.1186/1742-4690-5-7

**Published:** 2008-01-25

**Authors:** Amit Kapoor, Beth Shapiro, Robert W Shafer, Nancy Shulman, Soo-Yon Rhee, Eric L Delwart

**Affiliations:** 1 Blood Systems Research Institute, San Francisco, CA 94118, USA; 2 University of California San Francisco, CA, USA; 3 Henry Wellcome Ancient Biomolecules Centre, Dept of Zoology, Oxford University, Oxford, UK; 4 Division of Infectious Diseases, Department of Medicine, Stanford University Medical Center, Stanford, CA, USA

## Abstract

**Background:**

Combination anti-viral therapies have reduced treatment failure rates by requiring multiple specific mutations to be selected on the same viral genome to impart high-level drug resistance. To determine if the common protease inhibitor resistance mutation L90M is only selected once or repeatedly on different HIV genetic backbones during the course of failed anti-viral therapies we analyzed a linked region of the viral genome during the evolution of multi-drug resistance.

**Results:**

Using L90M allele specific PCR we amplified and sequenced gag-pro regions linked to very early L90M containing HIV variants prior to their emergence and detection as dominant viruses in 15 failed salvage therapy patients. The early minority L90M linked sequences were then compared to those of the later L90M viruses that came to dominate the plasma quasispecies. Using Bayesian evolutionary analysis sampling trees the emergence of L90M containing viruses was seen to take place on multiple occasion in 5 patients, only once for 2 patients and an undetermined number of time for the remaining 8 patients.

**Conclusion:**

These results indicate that early L90M mutants can frequently be displaced by viruses carrying independently selected L90M mutations rather than by descendents of the earlier mutants.

## Introduction

High rates of human immunodeficiency virus (HIV) replication and mutation in vivo results in the continuous generation of genetic variation [1]. HIV within a patient is therefore present as a mixture of related but distinct genetic variants collectively referred to as a quasispecies. HIV variants in different anatomical locations of the same individual also frequently differ possibly reflecting adaptation to local cellular environments, difference in immunological pressures and/or founder effects of tissue colonization [2,3]. Differences in the strength of anti-retroviral therapy selective pressure in different tissues and cell types may also contribute to the uneven distribution of drug resistance variation in vivo [4].

HIV protease inhibitors impair the maturation and resulting infectivity of viral particles leading to a rapid decline in plasma viremia as the major virus producing cells are depleted by viral cytopathic effects and/or immune responses. Different amino acid substitutions in the viral protease region are tightly associated with reduced sensitivity to protease inhibitors and rebounding viral loads. These mutations may also emerge in a sequential order [5-8]. Primary drug resistance mutations that alone confers moderate resistance such as V82A and L90M are initially selected followed by the addition of secondary mutations often located outside of the active site of the PR, such as L10I, M36I, M46I, L63P, or A71V leading to higher levels of resistance [9,10].

In addition to protease inhibitor resistance mutations in the protease gene, HIV protease cleavage site mutations can also be selected to compensate for reduced enzymatic activity against the wild-type cleavage sites [11-13].

Evolution of protease inhibitors resistance has been studied using mathematical models as well as longitudinal sequence analysis of HIV in vivo [9,14]. Such studies confirmed the expected presence, prior to therapy, of very low level of drug resistant mutants [15]. Both secondary protease resistance mutations and protease cleavage site mutations have been detected prior to protease inhibitor selection treatment [16]. The usually negative consequence of such drug resistance mutations on viral replicative fitness (in the absence of anti-retroviral therapy) is likely to keep the pre-treatment frequency of drug resistant mutants low [17]. Early during sub-optimal anti-retroviral therapy, weakly drug resistant viruses are therefore selected followed by the accumulation of further drug resistance mutations resulting in high-level drug resistance. The genetic characteristics of selected drug resistant variants in vivo has been longitudinally analyzed after these variants have reached a significant proportion of the plasma population using direct PCR sequencing methods [18-23]. Technically more demanding has been the analysis of the early stage of drug resistant mutant selection when selected mutants are still present at a very low frequency within the dominant drug sensitive viral quasispecies. Several studies have reported the emergence of previously minority variants carrying drug resistance mutations to dominate the later quasispecies and the frequent occurrence of viral recombination [24-26]. In this study we genetically characterized protease inhibitor resistant variants carrying the protease L90M mutation before they reached readily detectable frequencies (i.e. using direct PCR population sequencing) in patients failing salvage antiretroviral therapies. L90M is one of the most common protease-inhibitor resistance mutations and is selected primarily by the protease-inhibitors saquinavir, nelfinavir, and indinavir, at least one of which was received by each of the patients in this study. The L90M mutation, which is not located near the enzyme' s active site, is thought to displaces L24, which is adjacent to the catalytic residue D25, reducing the volume of the substrate cleft or protease dimer stability decreasing susceptibility to most protease inhibitors [27-29]. L90M allele specific amplification and sequencing of the resulting PCR product allowed us to compare an upstream linked region of early minority L90M variants with those of co-replicating dominant L90 viruses and to the L90M viruses which later came to dominate the plasma quasispecies [9,13,30]. Using Bayesian evolutionary analysis to sample alternative phylogenetic trees we determined that in a significant fraction of patients the early and later L90M carrying variants appear to have distinct origins, and therefore to have been originally selected on different genetic backbones.

## Results

### Selective amplification of variant carrying L90M mutation

PCR primers were designed and tested to specifically amplify variants carrying the protease drug resistance mutation L90M present at low frequencies within quasispecies dominated by wild type viruses. We targeted the protease L90M mutation as a frequently selected primary drug resistant mutation providing measurable levels of resistance to all currently approved protease inhibitors. The L90M mutation being located near the extremity of the protease gene also allowed linkage of early L90M mutations with other upstream protease polymorphisms. PCR primers located upstream of protease were also used to amplify a region of gag including multiple protease cleavage sites [13]. A protocol was first designed and tested for the specific amplification of minority L90M mutants starting from PCR products generated using generic gag-pro nested PCR (see material and methods).

**Figure 1:**
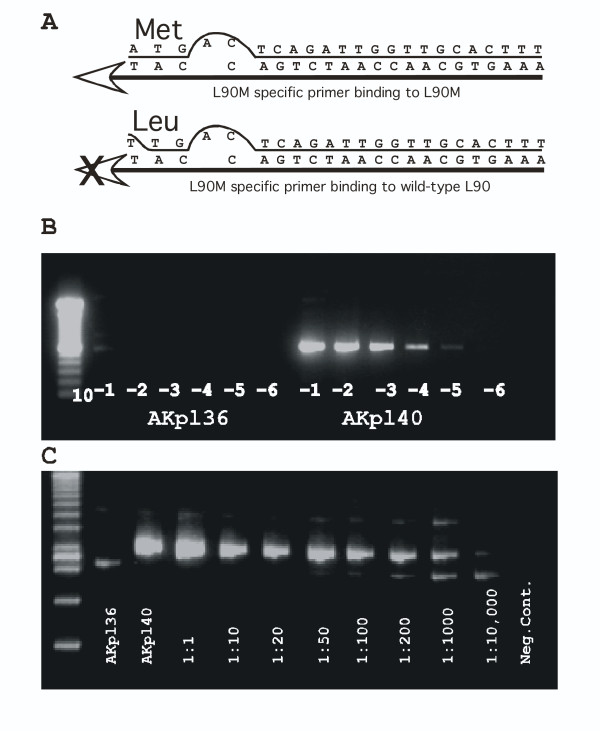
Design and test of the L90M allele-specific PCR protocol. A. Alignment of AK90m primer with mutant L90M and wild-type L90 region of protease gene (subtype B consensus). B. PCR amplification using L90M specific primer of serial log dilutions of L90 (AKp136) and L90M (AKp140) plasmid derived generic gag-pro amplicons. C. Sensitivity of L90M allele specific PCR using serial dilution of AKp140 derived amplicon in a constant amount of AKp136 derived L90 amplicon. Ratio of L90M to L90 amplicons DNA is shown

In order to measure the specificity of our L90M targeting PCR primer we first tested two plasmids (pAKL90 and pAK90M) encoding the w.t. L90 and mutant L90M codons respectively. The L90M specific primer AK90m was designed with its three 3' end nucleotides complementary to the L90M methionine codon (ATG). The 4^th ^complementary base of that primer (relative to HIV) was deleted and a 5^th ^base resulting in a mismatch was used (Fig. 1A). The next AK90m nucleotides were complementary to the HIV subtype B consensus sequence. Relative to other L90M targeting primers tested the deletion of the 4^th ^base and mismatching of the 5^th ^base significantly reduced background annealing to the w.t. L90 codon (TTG)(data not shown).

Ten-fold dilutions of generic gag-pro PCR products derived from plasmids pAKL90 and pAK90M were made using generic gag-pro 2^nd ^PCR round primers (AKG2 and EDPR4)(see materials and methods). These PCR product log dilutions were then tested using the L90M specific primer pair (AKG3 and AK90m). Up to 10^4 ^fold dilutions of the L90M generic gag-pro PCR products could be amplified while none of the L90 generic gag-pro PCR products was amplified (Fig 1B). To further evaluate the discriminatory capability of the L90M specific primer AK90m and more closely mimic conditions using clinical samples serial dilutions of the L90M PCR products were mixed with a constant amount of L90 PCR products. The presence of L90M DNA could still be detected after a 1000 fold dilution while the L90 DNA target generated only a shorter non-specific fragment at the highest L90M dilutions (Fig. 1C). To confirm that the amplicons generated from the highest pAK90M PCR DNA dilutions (1:200 and 1:1000) were indeed derived from the minority L90M variant they were purified and directly sequenced. Sequencing confirmed, through the detection of all 15 internal mutations distinguishing pAK90M from pAKL90, that the L90M specific primers had specifically amplified the L90M variant at the highest dilutions.

### Sequence analysis of clones for confirmation of minority population genotype

To further substantiate the specificity of our L90M specific amplifications we analyzed the second PCR round generic gag-pro PCR products from two plasma samples in which the L90M variants were detected only using L90M specific primers (i.e. not by direct sequencing of the generic PCR products). Generic gag-pro amplicons from patient samples 608 (03/98) and 1391 (05/99) were subcloned into E. coli plasmids and the presence of the L90M mutation determined using the L90M specific primers. 150 insert containing E. coli colonies (i.e. white) from each subcloning were replica plated and the presence of L90M in their inserts tested by colony PCR (see materials and methods). Two colony PCRs from 608 and one from 1391 were L90M positive. These plasmids were purified and the presence of the L90M mutations confirmed by plasmid insert sequencing. The sequence of the L90M plasmid subclones were also compared to those derived by sequencing the L90M specific amplicons derived from the generic gag-pol PCR. The sequence polymorphisms distinguishing the L90M specific amplicons from their respective majority population sequences were similar to those detected in the L90M positive plasmid subclones (data not shown). These results further support the specificity of detection of minority L90M variants using our allele specific PCR protocol.

### Protease gene evolution

To analyze the early evolution of L90M longitudinally collected plasma samples from 45 patients who developed L90M (as detected by direct PCR population sequencing) were obtained. Generic gag-pol amplification products from plasma samples available from earlier time points (negative for L90M by direct PCR population sequencing) were then tested using the L90M specific primers. The early presence of low frequency L90M variants was detected in fifteen of the forty-five patients. For nine patients only a single time point showed the presence of low-frequency L90M mutants. From these nine patients three sequences were derived. Direct PCR population sequencing of the generic gag-pol amplicons was used to determine the consensus sequences of both the early dominant w.t. and the later dominant L90M populations. The later time point generic gag-pro sequences invariably confirmed the dominance of the L90M mutation which was detectable by direct population sequencing while the earlier time point only showed w.t. codon 90 by direct population sequencing as expected from the patient selection criteria (see materials and methods). The early time point amplicons generated using the L90M specific primer were also directly sequenced to determine the consensus sequence of these minority L90M populations. Intermediate time points were available for another 6 patients and the generic gag-pro and L90M specific PCR products, when generated from these samples, were also directly sequenced.

### Bayesian analysis for monophyletic origin of L90M carrying variants

To determine whether it was appropriate to include all sequences in a single analysis and to test for recombination in the data set, we conducted a Phi test [31], as implemented in SplitsTree4 [32]. The Phi statistic tests for genealogical correlation between neighboring sites (which is negatively correlated with rate of recombination) using a modified pair-wise compatibility approach [31]. Use of SplitsTree v 4 found no evidence for recombination in the data set (p = 0.17).

**Figure 2:**
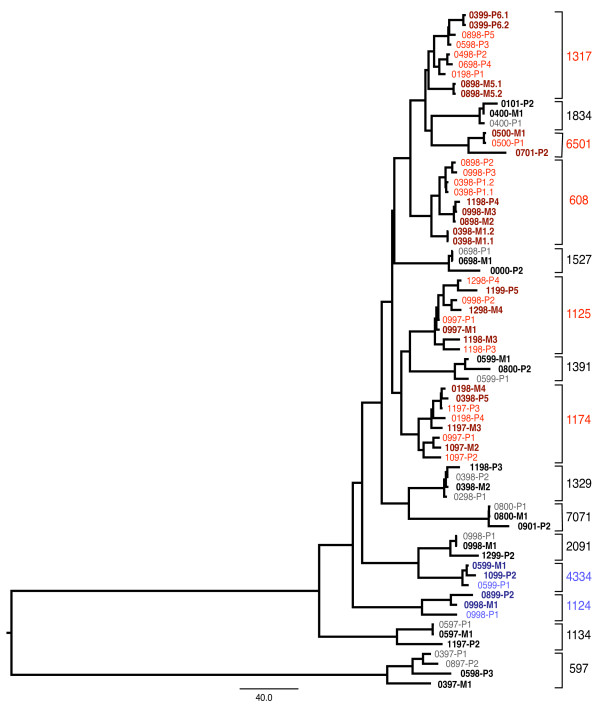
Summary tree showing the evolutionary relationships between all of the sequences used in this analysis, showing median node heights derived from 9000 posterior trees. Labels on the tips first show the sampling date in month and year and whether the generic (P for population) or L90M specific (M for mutant) PCR primers were used to generate the sequenced amplicons. The following number (1 through 6) represents the order of the bleeds analyzed. Patient numbers are given in the margin. Sequences that have the L90M mutation are indicated in darker bold fonts. Each patient forms a monophyletic cluster with 100% Bayesian posterior probability support (in 100% of posterior trees). Posterior support values for the monophyletic relationships of the sequences with the L90M mutation are given in Table 1. Patients with sequences showing evidence for multiple origins of L90M are shown in red, those with a single L90M origin in blue and those with an inconclusive number of L90M origins in black.

Fig 2 shows a summary of the phylogenetic relationships between the sequences derived in this study, in which each patient forms a well-supported monophyletic clade thereby excluding the possibility of contamination or sample mix-up. In order to investigate whether the two or more sequences carrying the L90M mutation (the early minority and the later dominant variants) were originally derived from a single virus within each patient, we investigated the probability that the L90M associated sequences formed a monophyletic clade with respect to sequences lacking the L90M mutation within each patient cluster. The probability of monophyly was calculated over 9000 trees drawn from the posterior distribution of the MCMC analysis, and is listed for each patient in Table 1. Our analysis indicated that the L90M mutations evolved on more than one occasion in at least 5 patients (p ≤ 0.05; patients 1174, 1317, 1125, 6501, 608). Alternatively, evidence for a single mutation event leading to both the early and the late L90M mutation was seen for two patients (p ≥ 0.95; patients 4334, 1124) while the phylogenetic evidence was inconclusive for the remaining eight patients (p: >0.05 to <0.95; patients 597, 1329, 7071, 1834, 1527, 1391, 2091, 1134).

**Table 1:** Probability of monophyletic origin for L90M sequences

**Patient**	**p(monophyly)**
**Multiple origins L90M**	

608	*0.05*
6501	*0.00*
1125	*0.00*
1174	*0.00*
1317	*0.00*

***Single origin L90M***	

1124	*0.99*
4334	*1.00*

***Inconclusive***	

597	*0.34*
7071	*0.29*
1134	*0.23*
1329	*0.14*
1391	*0.87*
1527	*0.30*
1834	*0.58*
2091	*0.18*

The probability that sequences containing the L90M mutation within each patient were derived from a single (p≥0.95) or multiple (p≤0.05) mutation events, given as the proportion of 9000 trees drawn from the posterior distribution of the Bayesian MCMC analysis in which the sequences with the L90M mutations formed a monophyletic clade.

## Discussion

The high level of genetic diversity of HIV in vivo provides it with multiple strategies for evolving high levels of anti-viral drug resistance. The originally selected drug resistant mutants may accumulate further drug resistance protease and RT mutations in a step-wise fashion to increase their level of drug resistance, develop resistance to new anti-viral drugs and attenuate fitness costs imposed by these mutations. Longitudinally observed drug resistant populations may also have independent origins, having been initially selected on different genetic backbones. In order to further investigate this phenomenon we selectively amplified and then sequenced HIV variants carrying the frequently detected protease inhibitor resistance mutations L90M when these viruses were still only present as minority variants. We then compared these viruses with the L90M viruses from the same patients that later came to dominate their plasma quasispecies. Evidence was obtained that in a substantial subset of patients (5/15) the L90M mutations were selected on multiple occasions. The L90M variant that eventually emerged to readily detectable levels (i.e. using direct PCR population sequencing) could therefore be of different origin than the earlier replicating minority L90M viruses. In another subset of patients our analysis indicated that the later dominant L90M variants descended from the earlier minority L90M variant in a manner consistent with step-wise drug resistance mutations gradually accumulating on descendents of the original L90M virus. The remaining patients could not be confidently categorized into either group.

In vivo and in vitro studies have suggested an important role for protease cleavage site mutations in restoring replicative fitness of drug resistant HIV variants [33,34]. We found no significant evolution of these cleavage sites among the 15 patients analyzed here.

While the phylogenetic analysis was able to differentiate between monophyletic or multiple origins of the L90M variants in many of the patients, it appeared most powerful when multiple sequences were available from several time points. Four out of five of the subjects with multiple L90M origins four were sampled at ≥4 time points (the fifth patient 6501 was sampled twice). All patients with apparently monophyletic L90M origins were sampled ≤3 times. Increasing the number of sampling over the course of infection may therefore significantly improve the capacity of such phylogenetic analyses to detect multiple origins of a drug resistance mutation.

It is also noted that HIV recombination within a patient, which is known to be commonplace in HIV-1 and is currently not possible to incorporate in a BEAST analysis, may also affect the resulting reconstructions. Recombination may rapidly shuffle neighboring sequence fragments obscuring the origin of different drug resistant variants. Indeed such recombination has been recently demonstrated to occur in vivo [25,26] as anticipated from the common detection of cell co-infected with multiple variants [35]. The Phi test used here works on the principle that in the presence of recombination, sites that are nearer to each other in sequence space should have greater compatibility with each other than will sites that are distant from each other [31]. The statistical significance of genealogical correlations of adjacent sites is then evaluated using a permutation test, in which the null hypothesis of no recombination would result in no effect on correlation of adjacent sites after permutation (as all sites share the same history). The test is particularly appropriate for this data set as it does not assume a single population, is powerful to detect recombination regardless of population and demographic history, mutation rate and rate of recombination, and has been shown to accurately distinguish between recent mutations and recombination even with closely related sequences. While recombination was not detected it remains possible that more frequent sampling or the analysis of clonal sequence data rather than population consensus sequences may have revealed a greater level of recombination.

The detection of a polyclonal origin for the commonly selected L90M protease mutation was anticipated given the constant generation of HIV mutants. Selection of some drug resistance mutation has been shown as early as the initial phase of viremia control in patients undergoing suppressive therapy [36]. This study shows that a common drug resistance mutation can frequently have multiple origins.

## Methods

### Patient Samples

The plasma samples of HIV infected persons undergoing direct PCR sequencing for drug resistant genotyping at Stanford University Hospital Diagnostic Virology Laboratory meeting the following criteria were selected for this study: (i) Two drug resistance genotypes were performed before or following virologic failure on a protease inhibitor containing regimen; and (ii) the first and second genotype performed showed complete absence followed by the presence of the primary protease inhibitor resistance mutationL90M using direct PCR population sequencing. Available viral loads and antiretroviral drug regimen are shown (see Additional file 1). The study was approved by Stanford University Hospital and the University of California San Francisco committees on human research.

### Viral RNA isolation and RT-nPCR

Viral RNA was extracted from 280 μl of patient's plasma using QIAGEN viral RNA extraction kit and eluted in 50 μl of RNase-free water. 15 μl of extracted RNA was reverse transcribed using 5 pmol of primer EDPR2 (TTGTTTAACTTTTGGGCCATCC [HXB2 positions 2597 to 2618]) in a solution containing 2 μl of 10 mM dNTP at 95°C for 10 sec, followed by 65C for 5 min. 200 U of SuperScript II RNase H^- ^Reverse transcripatse, 5× first strand buffer, 30 U of RNase Inhibitor in final volume of 25 μl was kept on 42°C for 60 min, followed by 75°C for 15 min to inactivate the enzyme. Nested PCR using HIV-1 subtype B primers was then used to amplify a fragment encompassing seven HIV protease cleavage sites (CA/p2, p2/NC, NC/p1, NC/TFP, p1/p6, TFP/p6 and p6/protease) and all 99 amino acids of HIV protease gene (from HXB2 positions 1829 to 2577). First round of PCR was done using 10 μl of cDNA and primers AKG1 (GATGACAGAAACCTTGTTGGTCCA HXB2 positions 1736 to 2163]) and EDPR2 (TTGTTTAACTTTTGGGCCATCC HXB2 positions 2597–2618) generating a 882 bp product. Second round PCR used 1 μl of first round PCR product and primers AKG2 (GACAGCATGTCAGGGAGTAGG HXB2 positions 1829–1850) and EDPR4 (CTGGTACAGTTTCAATAGGACTAATGG HXB2 positions 2551 to 2577) generating a 748 bp product [22]. In text we refer to these primers as the first and second PCR rounds generic gag-pro primers. The final volume of each PCR reaction was 50 μl containing 10 pmol of each primers, 10 mM Tris-HCl pH 9.0, 50 mM KCl, 2.5 mM MgCl_2_, 0.1% triton-X100, 2.5 mM of each dNTP and 3.5 U *Taq *polymerase. PCR program consisted of 3 cycles at 94°C for 45 s, 57°C for 45 s, and 72°C for 1 min, followed by 27 cycles at 94°C for 30 s, 57°C for 30 s, and 72°C for 1 min, and final extension at 72°C for 5 min. Using pNL4-3 plasmid dilutions the sensitivity of this nested PCR was determined to be between 1 and 10 copies (data not shown). Second round PCR products generated with AKG2 and EDPR4 were purified using Quiagen PCR product purification kit and sequenced using primer EDPR4. Sequencing of the entire PCR products was on occasion prevented by the presence of variants of different length in the PCR product (due to the co-amplification of indel variants). When sequencing such PCRs the sequencing electrophoregram abruptly shows the presence of mixed bases at every nucleotide position following an insertion-deletion (indel) containing region. To allow sequencing beyond the region of length polymorphism these PCR fragment were sequenced from the other direction using primer AKG2 allowing us to generate the consensus sequence on both side of the indel. For patients 608 and 1317 both consensus sequences (with and without extra indel codon) are used in Figure 2 (taxa labels ending in .1 and .2).

### Selective amplification of minority variant carrying L90M protease mutation

The pGEM-T vector backbone plasmids pAKL90 and pAK90M each have a 748 bases long HIV fragment insert of the second round PCR product generated by the second PCR round generic gag-pro primers AKG2 and EDPR4. pAK90M has the L90M mutation (methionine codon 90 = ATG) while pAKL90 is wild type L90 (leucine codon 90 = TTG). pAKL90 and pAK90M also differ at 15 nucleotides positions within the fragment amplified by the L90M specific PCR.

Input DNA into the L90M specific PCR consisted of PCR DNA generated from either plasmids or from cDNA from clinical samples using the nested PCR generic gag-pro primers. Second round PCR DNA was purified using Quiagen PCR product purification columns, diluted 1:100 in water and 10 μl of dilution was used as input for the L90M selective PCR. The L90M specific primer was AK90m (AAAGTGCAACCAATCTGACCAT HXB2 positions 2520–2542) used together with AKG3 (ACCCGGCCATAAAGCAAG HXB2 positions 1853–1871) in a final PCR reaction volume of 50 μl containing 10 mM Tris-HCl pH 9.0, 50 mM KCl, 2.5 mM MgCl_2_, 0.1% triton-X100, 2.5 mM of each dNTP, 15 pmol of each primer and 3.5 U Hot start *Taq *polymerase. PCR cycling consisted of polymerase activation at 95°C for 15 min followed by 5 cycles at 95°C for 45 s, 50°C for 45 s, and 72°C for 1 min, and 10 cycles at 95°C for 30 s, 50°C for 30 s, and 72°C for 1 min, and final extension at 72°C for 5 min. L90M specific amplification products generated with AK90m and AKG3 were purified using Quiagen PCR product purification kit and sequenced using primer AKG3. When indels variants of different length were co-amplified, preventing complete sequencing from AKG3, the primer AK90m was also used to sequence from the other direction. Amplification of L90M variants from E. coli colonies was performed by touching a sterile toothpick to each colony and the toothpick then rubbed on the inside of a L90M specific PCR reaction mixture followed by only eight cycle of L90M specific PCR amplification (using AK90m and AKG3). The PCR products were analyzed by PAGE.

### Sequence analysis

PCR products of second round generic gag-pro amplicons were 748 nucleotides. The L90M specific amplicons were 689 nucleotides. 611 nucleotides (HXB2 position 1903 to 2513) were included in the phylogenetic analysis to accommodate nucleotide sequence unavailable from the shorter L90M specific amplicons and low quality sequence data immediately adjacent to the sequencing primer. Mixed bases at nucleotide position were recorded in direct sequencing when one minority electrophoregram peak was present at ≥40% of the total peak height. Genbank accession numbers are: EU380596–EU380664

Sequence data was generated from 15 patients. The number of sequences collected for each patient varied from three to nine depending on the number of time points available. Sequences were aligned using CLUSTAL W with all settings set to the default values, and checked by eye. Bayesian phylogenetic analysis was performed using the software BEAST v1.4 (Drummond AJ & Rambaut A (2006) BEAST v1.4, Available from [37]) that allows the incorporation of sampling times into the evolutionary model to inform the molecular rate. A relaxed molecular clock [38] was assumed, with a constant-size coalescent prior on the tree. The HKY+G_4 _model of nucleotide evolution was used, which allows for different rates of transitions and transversions and for rate heterogeneity along the sequence (four categories). Posterior distributions of parameters and trees were investigated using Markov chain Monte Carlo (MCMC) analysis, with samples from the posterior drawn every 2000 steps over a total of 20,000,000 steps, and the first 10% discarded as burn-in.

## Supplementary Material

Additional file 1 Longitudinal viral loads, anti-HIV treatments, and direct PCR population sequencing drug resistance genotypes of salvage patients. Salvage patient summaries show plasma HIV-1 RNA levels (on a log scale), direct PCR population sequencing drug resistance genotypes, and anti-retroviral treatment histories. Large square and circle symbols indicate cryopreserved samples available for the generic protease and L90M specific amplifications described in this study. Solid squares indicate samples in which L90M was detected as minority variants while no L90M variants were detected in empty square samples. Empty circle indicate samples in which L90M was the dominant variant as determined by direct PCR population sequencing. Small closed circles indicate samples for which only plasma HIV-1 RNA level determination and/or population-based drug resistance genotypic testing was done. Click here for file
