# Supplementary figures and images for: Multiple independent origins of a protease inhibitor resistance mutation in salvage therapy patients
Source: Retrovirology. 2008 Jan 25;5:7. doi: 10.1186/1742-4690-5-7 (PMC2265302; doi:10.1186/1742-4690-5-7)

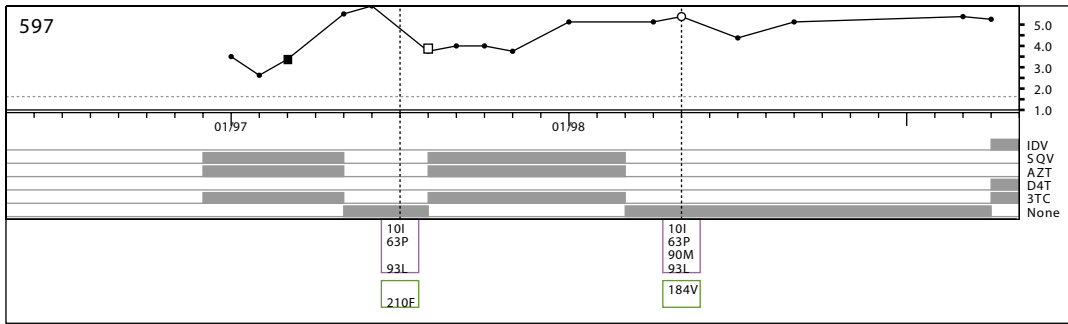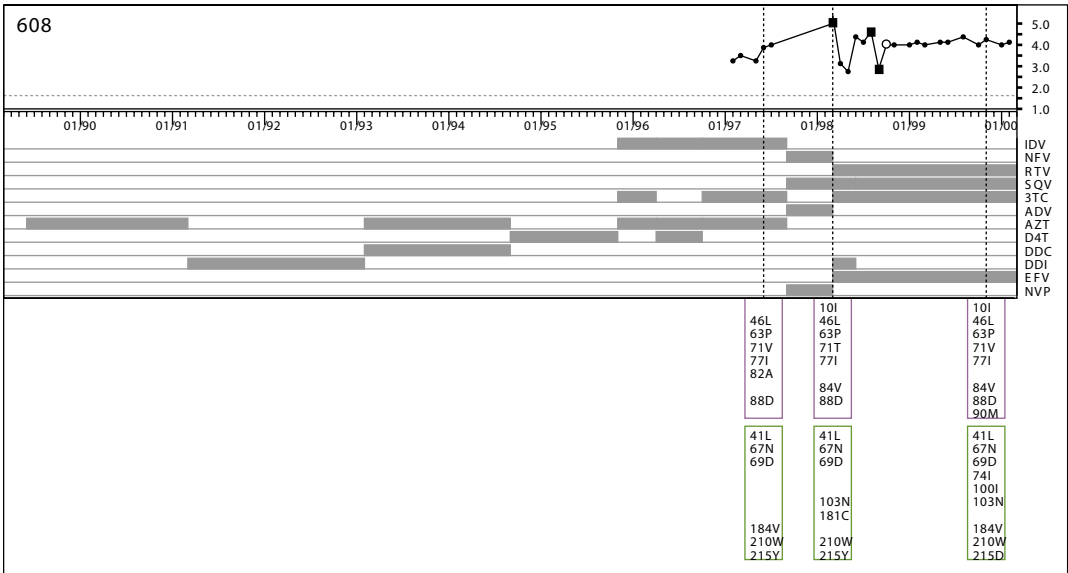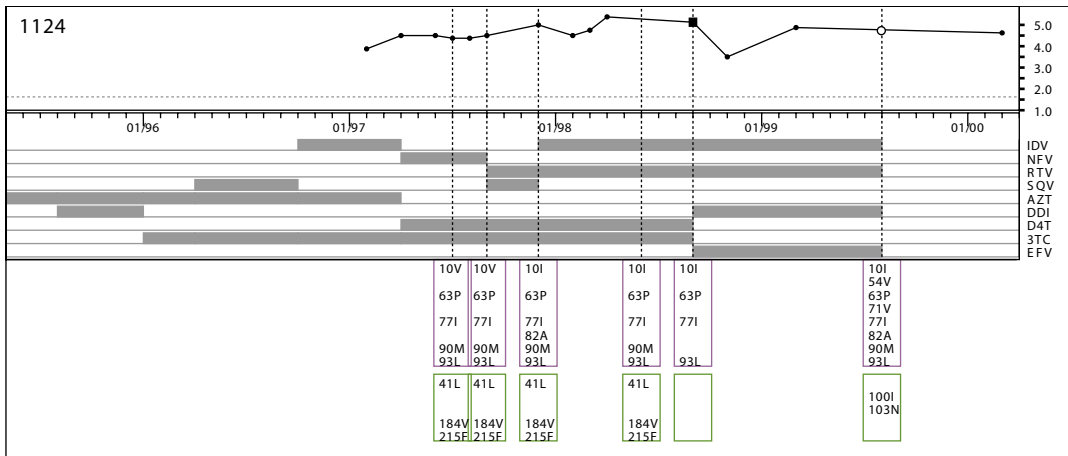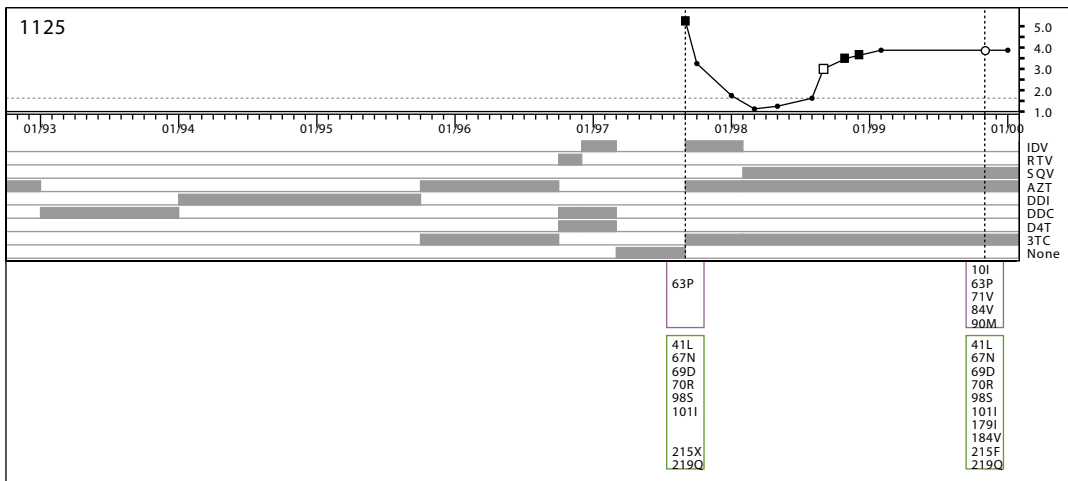

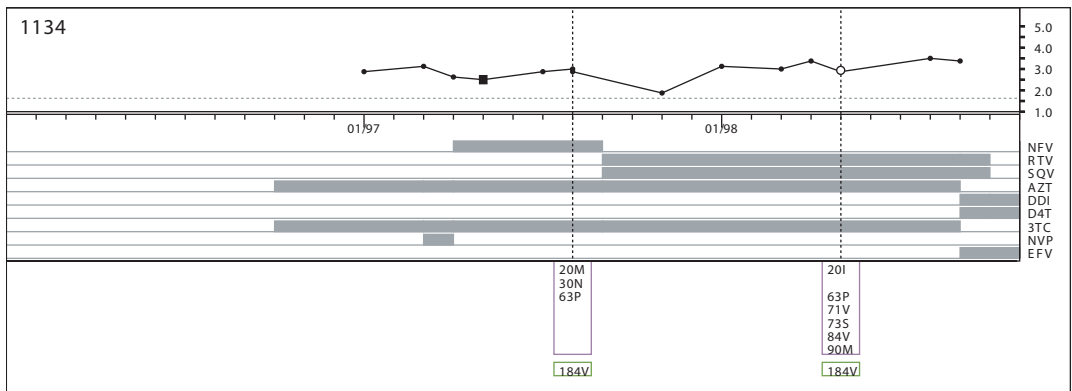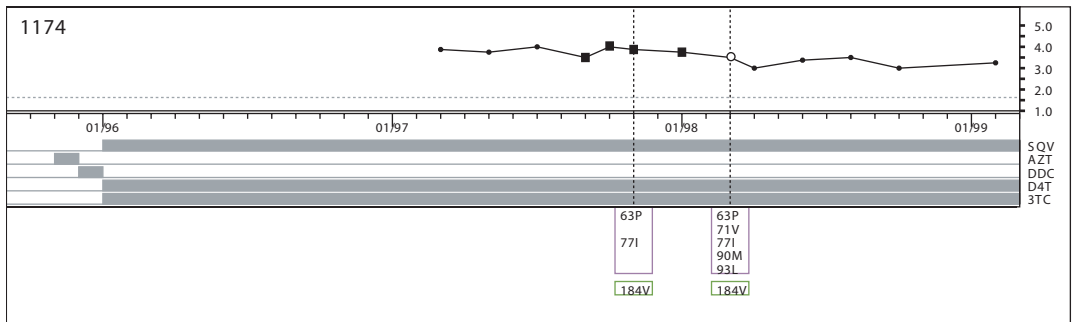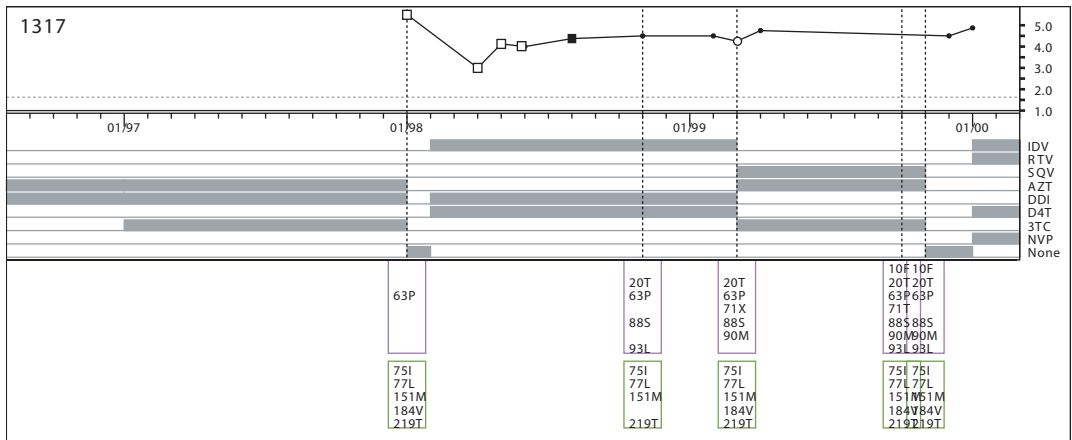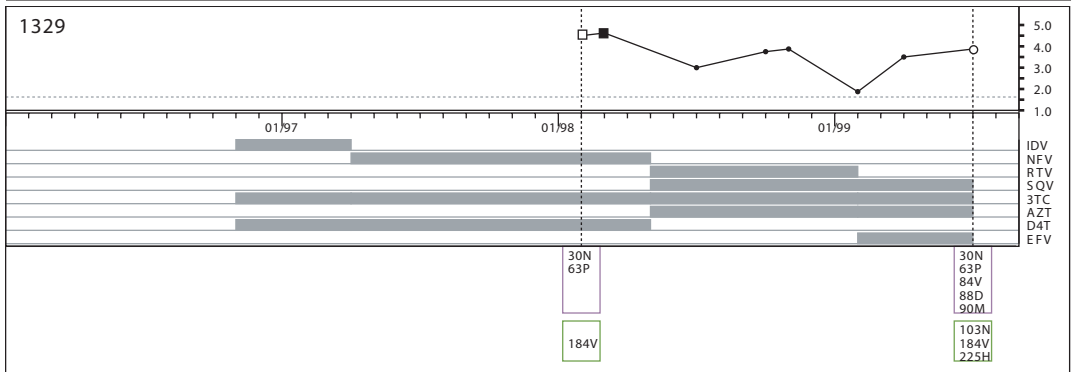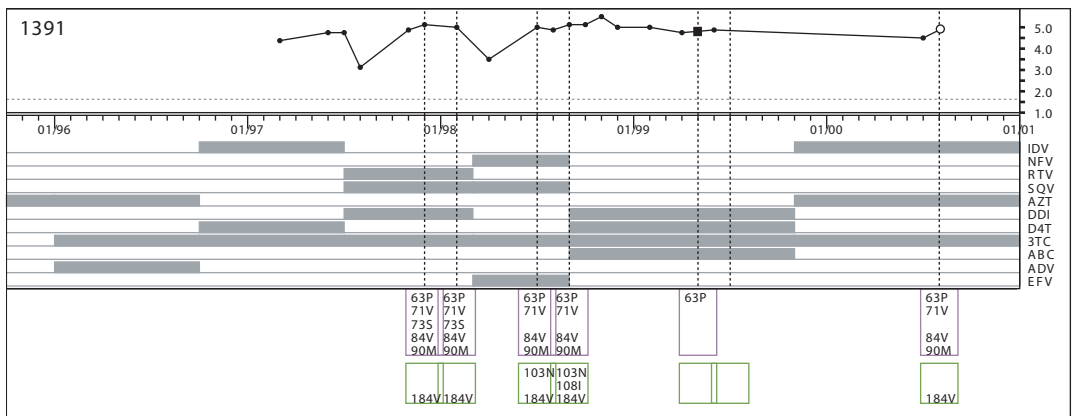

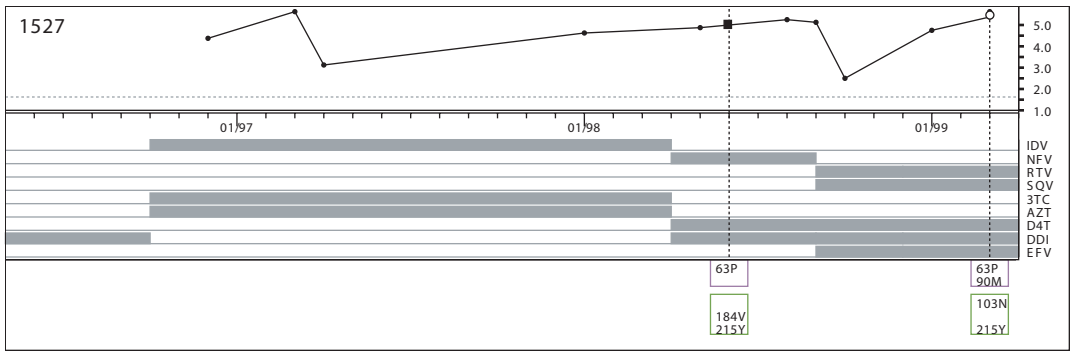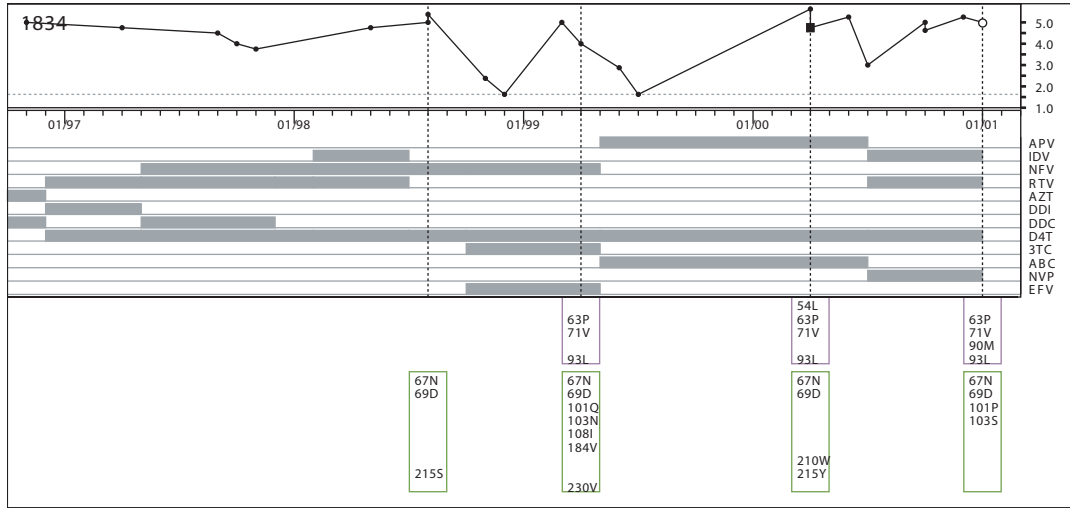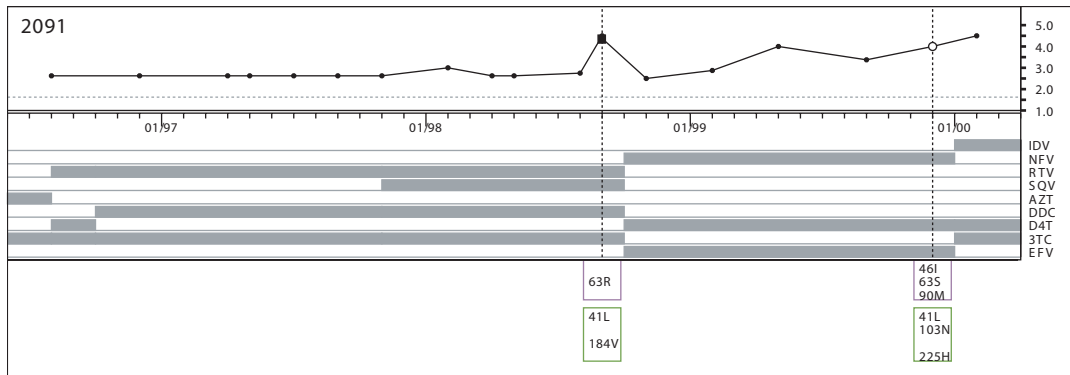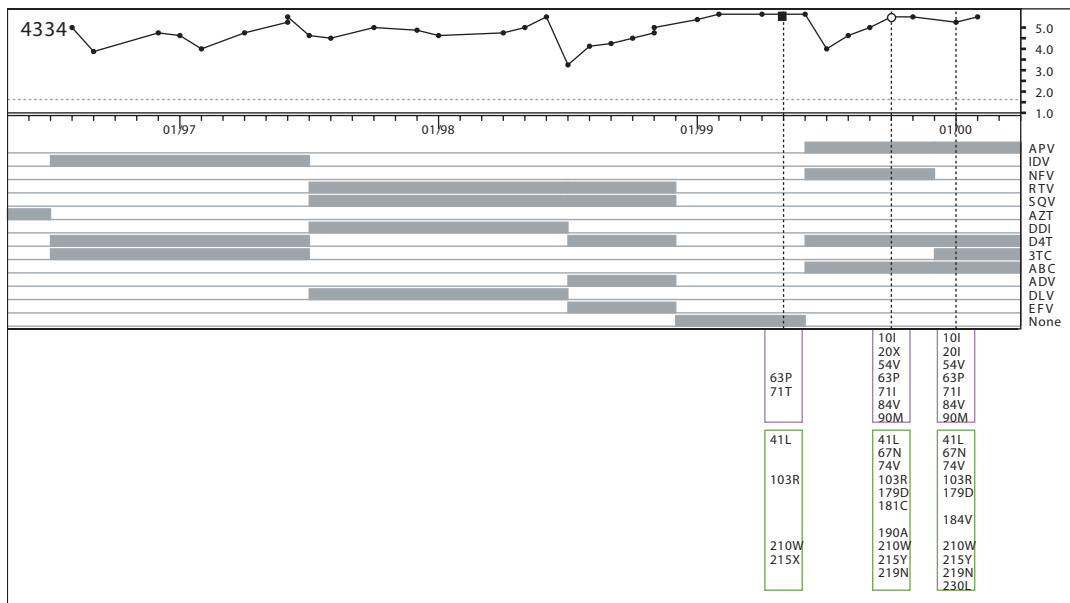

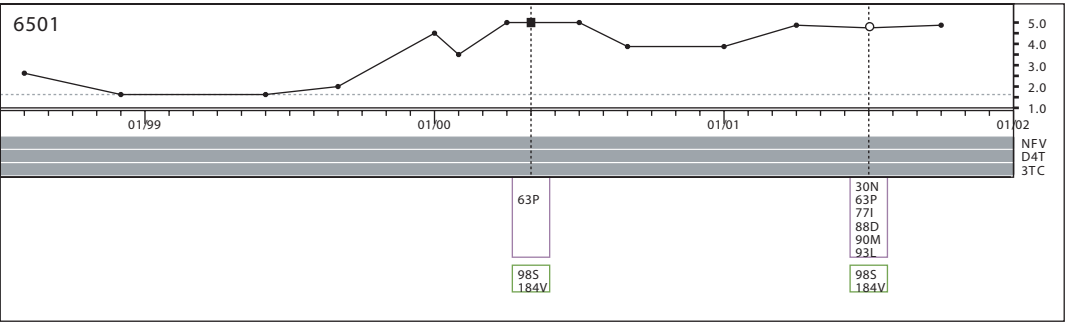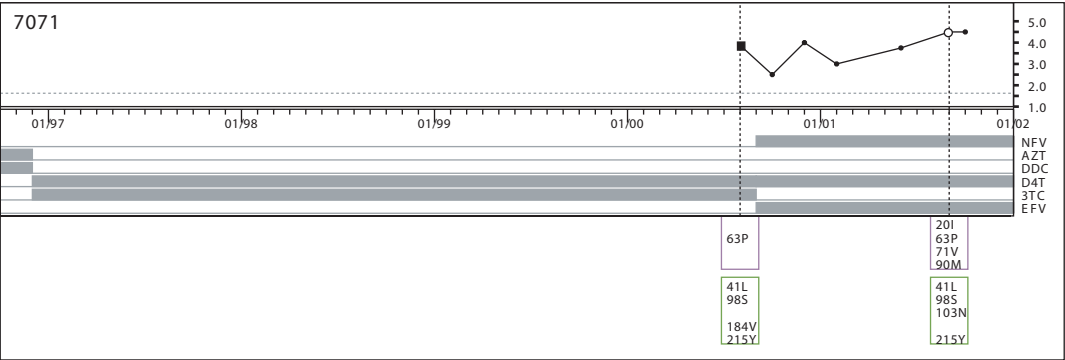

Supplement: Additional file 1 — Longitudinal viral loads, anti-HIV treatments, and direct PCR population sequencing drug resistance genotypes of salvage patients. Salvage patient summaries show plasma HIV-1 RNA levels (on a log scale), direct PCR population sequencing drug resistance genotypes, and anti-retroviral treatment histories. Large square and circle symbols indicate cryopreserved samples available for the generic protease and L90M specific amplifications described in this study. Solid squares indicate samples in which L90M was detected as minority variants while no L90M variants were detected in empty square samples. Empty circle indicate samples in which L90M was the dominant variant as determined by direct PCR population sequencing. Small closed circles indicate samples for which only plasma HIV-1 RNA level determination and/or population-based drug resistance genotypic testing was done. [file 1742-4690-5-7-S1.pdf]
